# Field postmortem examination practices in cattle production systems: a scoping review of knowledge, attitudes, training and barriers

**DOI:** 10.3389/fvets.2026.1968345

**Published:** 2026-10-15

**Authors:** Nathaly Vargas, Daniel Stuart, Elise Marshall, Nanny Wenzlow, Clinton Roof, Elpida Artemiou

**Affiliations:** 1 Texas Tech University School of Veterinary Medicine, Amarillo, TX, United States; 2 Texas Tech University Health Sciences Center, Amarillo, TX, United States

**Keywords:** cattle production system, field necropsy, knowledge and attitudes, postmortem examination, veterinary professionals

## Abstract

**Background:**

Postmortem Examination (PME), or necropsy is an important tool for diagnosing disease, determining cause of death, monitoring herd health and supporting epidemiological surveillance in cattle production systems. Despite its recognized value, PME is not routinely performed in the field.

**Objective:**

This scoping review aimed to synthesize the available evidence regarding knowledge, attitudes, barriers, and training needs related to field PMEs in cattle production systems.

**Methods:**

A scoping review was conducted following the methodological framework proposed by Arksey and O'Malley and further refined by Levac et al. Reporting of the review was guided by the Preferred Reporting Items for Systematic Reviews and Meta-Analyses extension for Scoping Reviews (PRISMA-ScR). Literature searches were performed in PubMed, Scopus, Embase, Web of Science, and Google Scholar, supplemented by manual searches and citation tracking. Studies were eligible for inclusion if they addressed field PMEs in cattle production systems and involved veterinarians, producers, farm workers, managers, or veterinary students. Data were extracted, tabulated, and synthesized descriptively.

**Results:**

A total of 988 references were identified. After removing 328 duplicate references, 660 records underwent title and abstract screening, and 521 were excluded, leaving 139 full text references for eligibility. Title and abstract screening and full-text review were completed independently by two reviewers. Inter-reviewer agreement was fair during title and abstract screening (Cohen's κ = 0.285) and high agreement during full-text review (Cohen's κ = 0.953). Twelve (12) studies met the inclusion criteria, exploring field PME training and reporting practices while identifying gaps and barriers across veterinary stakeholders.

**Discussion:**

The limited evidence identified in this review highlights a lack of standardized procedure for field PME performance and training practices that will support evidence-based decision-making for herd health.

**Conclusions:**

The reviewed literature consistently highlighted the value of field PMEs in terms of disease diagnosis and herd health management. Reported barriers included producer reluctance, time constraints, economic considerations, carcass disposal challenges, biosecurity concerns, and lack of training in PME procedures. Several studies also identified the lack of training and continuing education opportunities for veterinarians.

## 1 Introduction

Postmortem Examination (PME), or necropsy, is a valuable diagnostic procedure used to determine the cause of an animal's death, which plays a critical role in disease diagnosis, herd health management, outbreak investigation and control (1, 2).

In cattle production, PME is an essential procedure for disease monitoring and the identification of zoonotic outbreaks, by providing samples for laboratory confirmation as required to help safeguard both animal and human health (3). By determining the cause of death, veterinarians can monitor disease trends, evaluate the effectiveness of vaccination and treatment protocols, and implement evidence-based herd health management strategies (2). High mortality rates on a farm are a significant welfare concern, as they may indicate underlying disease, injury, inadequate nutrition, management deficiencies, or ineffective treatment protocols. PME findings can inform herd health management interventions at both the farm and national levels (1, 4).

Livestock mortality results in substantial economic losses for producers, not only through the loss of the animal's market sale-value, but also from costs associated with treatments, labor, carcass disposal, and replacement (2). In dairy systems, these losses include forgone milk production, whereas in beef production they include the loss of market value and prior investments in feeding and animal health. The economic impact is particularly relevant in the intensive cattle production region, such as Texas, where dairy and feedlot operations represent an important component (5–7).

PMEs can be performed either in a diagnostic laboratory or in the field, depending on available resources, circumstances surrounding the death, and biosecurity considerations, particularly if a highly pathogenic or zoonotic case is suspected. Field PMEs offers the advantage of rapid assessment because the carcass can be examined immediately after death, minimizing the effects of tissue decomposition that may compromise diagnostic accuracy. Timely diagnosis is essential for implementing herd management decisions, including quarantine, vaccination, or treatment of at-risk animals (2, 8). For cattle, transporting a carcass to a diagnostic laboratory can be expensive and logistically challenging because of its size and weight. In addition, carcass movement may be subject to state animal health regulations and biosecurity requirements, particularly when a reportable disease is suspected. These logistical, financial, and regulatory constraints can delay or prevent laboratory submission (9).

Conducting a PME on the farm also enables the veterinarian to directly evaluate the animal's environment, and management practices as well as potential risk factors that may have contributed to the death, providing valuable context that is often not available in a laboratory setting. Furthermore, when an infectious or biosecurity threat is suspected, immediate control measures can be taken, including isolating the carcass, quarantining exposed animals, and implementing enhanced biosecurity measures without delay. Beyond individual animal case investigations, field PMEs serve as an important component of routine herd health surveillance supporting evidence-based management decisions and helping prevent future losses.

Despite the recognized diagnostic value of field PMEs, several barriers limit their implementation reported. In one study, the primary reason that discouraged practitioners from performing a PME was the producer's refusal to authorize the service (69%), followed by time (44%), cost (38%), and carcass disposal (27%) (2). A lack of experience and training among farm personnel can delay the detection of mortality or the request for a professional PME. Furthermore, veterinarians reported deficiencies in their own training particularly in specialized PME techniques, while also noting difficulties with the emotional aspects of death (2). Lastly, while veterinarians are acutely aware of the risks associated with performing field PMEs, approximately 20% declined to perform on-farm PMEs because of concerns regarding biosecurity risks, while others that do perform them often implement extensive biosecurity measures to minimize these risks (10).

In field cattle operations, PMEs may involve personnel with varying levels of training and professional responsibilities. While veterinarians are responsible for interpretating findings and establishing diagnoses to inform clinical and herd level decision-making, other stakeholders, including farm personnel, managers, and producers may participate in conducting and documenting PMEs, reporting observations, and communicating relevant information to veterinarians. Such involvement may be particularly important under field conditions, where limited veterinary availability, distance from diagnostic laboratories, challenges associated with transporting large carcasses, cost, and other logistical constraints can limit access to veterinary diagnostic services. Consequently, bovine PMEs may be performed on-farm, and supplemented by appropriate sample collection and laboratory testing when needed (11, 12).

### 1.1 Gap

Although field PMEs are widely recognized as a valuable diagnostic tool, information regarding stakeholder knowledge, perceptions, skills, training, and barriers to implementation remains limited. To our knowledge, this is the first scoping review to synthesize this evidence. Accordingly, this review aimed to explore the existing knowledge, perceptions, practical competencies, training needs, barriers, and implementation practices related to PMEs among veterinary professionals and other relevant stakeholders in food-producing cattle systems including dairy, feedlot and cow-calf operations.

## 2 Methods

### 2.1 Methodological framework

This scoping review was conducted following the methodological framework proposed by Arksey and Ómalley (13) and further refined by Levac et al. (14). The review was reported in accordance with the Preferred Reporting Items for Systematic Reviews and Meta-Analyses Extension for Scoping Reviews (PRISMA-ScR) (2018). A comprehensive search strategy was developed using Boolean operators (“AND”, “OR”) to systematically combine key words related to field PME (necropsy), cattle systems (cattle, dairy, feedlot, cow-calf operations), relevant stakeholders (veterinarians, farmworkers, managers, veterinary students), and domains of interests including knowledge, perceptions, skills, training and barriers.

### 2.2 Search strategy

A comprehensive electronic literature search was conducted across five primary search sources: PubMed, Embase, Web of Science, Scopus, and Google Scholar. Supplementary search methods included hand searching, citation searching, EBSCO Discovery, and AI-assisted search functions available through Scopus and Embase. The PubMed search strategy combined Medical Subject Headings (MeSH) and free-text terms organized in four concept groups: (1) postmortem examination or necropsy, (2) cattle production systems, (3) relevant stakeholders, and (4) knowledge, perceptions, barriers, and training. Boolean operators (“AND”, “OR”) were used to combine these search terms within and across concept groups. This strategy was adapted as appropriate for the remaining databases, Searches were limited to studies published between 2000–2025 and to studies available in English.

### 2.3 Inclusion and exclusion criteria

The inclusion and exclusion criteria are summarized in Table 1. Eligible studies were peer-reviewed primary research articles published in English, between 2000 to 2025. Studies focused on cattle production systems, including dairy, feedlot and cow calf operations, and addressed field PMEs conducted in on-farm or production settings. Included studies examined knowledge, perceptions, attitudes, practical competencies, training needs, barriers, or implementation practices related to PME practices among relevant stakeholders, including farm workers, producers, managers, and veterinarians. Studies describing or evaluating the implementation of PMEs in real-world production environments were also eligible. Qualitative, quantitative, and mixed-methods study designs were included, encompassing surveys, interviews, and observational approaches. Studies including multiple livestock species were eligible when cattle represented a relevant component of the study population and cattle-specific findings related to field PME could be identified or extracted. Studies focusing exclusively on non-cattle species were excluded. Studies were excluded if they focused on laboratory-based PMEs rather than field or on-farm settings. Additionally, studies not directly related to PME practices within cattle production systems were not considered eligible.

**Table 1:** Inclusion and exclusion criteria of the reviewed literature.

Category	Inclusion	Exclusion
Population	Veterinarians, veterinary students, veterinary pathologists, producers, farm workers, and herd managers	Populations unrelated to cattle PME
Species	Cattle, including dairy cattle, beef cattle, feedlot cattle, and calves	Companion animals, wildlife, non-cattle livestock, humans
Setting	On farm, field production settings	Laboratory-based PME only
Focus	Knowledge, perceptions, barriers, attitudes, training, and implementation of PMEs	Studies unrelated to PME in cattle production
Study type	Peer-reviewed primary research; qualitative, quantitative, mixed methods	Diagnostic case studies
Language	English	Other languages
Year	2000–2025	Outside date range

### 2.4 Search outcomes

Two reviewers independently completed title and abstract screening and full-text reviews. Inter-reviewer agreement was fair during title and abstract screening (Cohen's κ = 0.285) and almost perfect during full-text review (Cohen's κ = 0.953). The lower agreement observed during title and abstract screening (Cohen's κ = 0.285) reflected differences in the initial interpretation of broad eligibility criteria, particularly for studies in which PME was not the primary study objective but potentially contained relevant evidence. Disagreements were discussed between reviewers and resolved by consensus before full-text assessment. The substantially higher agreement during full-text review (κ = 0.953) reflected the greater information available for applying the eligibility criteria.

### 2.5 Data extraction

Data extraction was performed independently by three of the authors (NA, EM, and EA) using a standardized approach. The information was extracted from each selected study, including authors, publication year, country in which the study was conducted, study objectives, study design, population and sample characteristics, data collection methods, data analysis, key outcomes, reported limitations, and relevance to field PME and veterinary practice (see Tables 2, 3).

**Table 2:** Characteristics of the references included in the scoping review.

References	Country	Study design	PM examiners/ participants	Count	Main findings
Alhaji and Odetokun (18)	Nigeria	Cross-sectional survey	Veterinarian	137	Veterinarians demonstrated greater knowledge of zoonoses and perceived a higher level of risk than para-veterinarians. Use of PPE during high-risk procedures was reported to be inconsistent.
			Para-veterinarian	392	
Alvåsen et al. (10)	Sweden	Pilot observational study	Veterinarians	10	Remote digital PME demonstrated good agreement with field PME findings and was identified as a feasible tool for supporting disease diagnosis and surveillance.
McConnel et al. (11)	United States	Observational study	Veterinarians	4	Standardized PME protocols facilitated the identification of multifactorial causes of death and highlighted the importance of systematic PMEs for accurate diagnosis and disease surveillance.
Abuelo and Mann (17)	United States	Cross-sectional survey	Veterinarians	164	The need for continuing education varies according to professional experience, with participants expressing a preference for hybrid learning models combining online instruction with hands-on practical training.
Salopek et al. (2)	United States	Cross-sectional survey	Veterinarians	52	PME was widely considered a valuable diagnostic tool; however, implementation was hindered by producer reluctance and limited confidence in lesion interpretation.
Gilbert et al. (27)	United Kingdom	Cross-sectional survey	Farmers	69	Veterinary involvement strengthened disease surveillance efforts, while producer attitudes influenced the implementation of PMEs.
			Veterinarians	10	
Kennedy et al. (28)	Ireland	Cross-sectional survey	Farmers	1,179	Laboratory sample submissions informed herd health management decisions. Logistical barriers, including travel distance, time constraints, and labor needs, limited sample submission.
Cherry et al. (29)	United States	Cross-sectional survey	Veterinarians	469	Knowledge of zoonotic diseases varied, and awareness of standard veterinary prevention and control precautions remained limited despite widespread PPE availability.
Thomsen et al. (30)	Denmark	Observational study	Farmers	69	PMEs improved identification of causes of death and revealed discrepancies between farmer-reported assessments and PME findings.
Davies et al. (12)	Canada	Experimental study	Veterinarians	15	Real-time pathologist assistance increased the rate of definitive diagnoses and improved sample collection practices and diagnostic confidence.
			Veterinary pathologists	4	
Lemos et al. (19)	Brazil	Observational study	Veterinarians	95	Combining tissue samples with photographic images improved diagnostic accuracy compared with photographic images alone.
Mijares et al. (31)	United States	Cross-sectional survey	Veterinarians	47	PME was considered an important diagnostic tool for BRD, and veterinarians identified priorities for diagnostics, treatment, and future research.

**Table 3:** Characteristics of the field postmortem examinations reported in the included studies.

References	PME setting/type	PME conducted by	Unit reported	Count
Alvåsen et al. (10)	On-farm remote digital necropsy	Trained veterinarians; images interpreted remotely by a bovine veterinary practitioner	Remote digital necropsies	15
McConnel et al. (11)	On-farm PME	Veterinarians	PMEs	174
Thomsen et al. (30)	PME at an incineration facility	Veterinarian with specialist training in pathology	PMEs	79
Davies et al. (12)	Unassisted on-farm PME	Veterinarians	PMEs	32
	Real-time pathologist-assisted on-farm PME	Veterinarians with remote assistance from veterinary pathologists	PMEs	26
Lemos et al. (19)	Diagnostic laboratory PME	Specialized laboratory veterinary team	PMEs	45
	Field PME with laboratory sample submission	Veterinarians	PME-derived sample submissions	39

### 2.6 Data analysis

Data were analyzed using a descriptive and narrative synthesis approach, and summarized according to study characteristics, including title, authors, publication year, country where study was performed, study objective, study design, population/ sample, methods, data analysis, key outcomes, limitations and relevance to postmortem examination (PME) practices.

## 3 Results

### 3.1 Characteristics of the included studies

The initial search identified a total of 988 records across multiple sources, consisting of PubMed (*n* = 259), Scopus (*n* = 235), Embase (*n* = 203), Web of Science (*n* = 196), Google Scholar (*n* = 34), hand searching (*n* = 33) (PubMed MeSH-specific searches, *n* = 12; Embase PICO Tool, *n* = 10; EBSCO Discovery, *n* = 11), citation searching (*n* = 15), and Artificial Intelligence (AI) tools (*n* = 13), including Scopus AI and Embase AI-based search engines.

After removing duplicates (*n* = 328), a total of 660 records remained for title and abstract screening. During the screening phase, 521 records were excluded because they were not relevant to the review objectives. A total of 139 full-text references were assessed for eligibility, and all identified studies were successfully retrieved. Following full-text review, 127 studies were excluded for reasons including focus on postmortem examination solely as a diagnostic tool (*n* = 67), focus on veterinary practice without specific evaluation of postmortem examinations (*n* = 16), assessment of technical skills other than postmortem examination (*n* = 14), focus on animal welfare (*n* = 7), focus on pathology curricula (*n* = 7), postmortem protocols without evaluation of relevant competencies or educational outcomes (*n* = 4), forensic or laboratory postmortem guidelines only (*n* = 4), focus on zoonotic pathogens and biosecurity without specific evaluation of postmortem examinations (*n* = 3), human autopsy (*n* = 1), canine postmortem examination (*n* = 1), equine postmortem examination (*n* = 1), human mental health (*n* = 1), and exotic animal disease and postmortem examination (*n* = 1).

Twelve (12) studies met the inclusion criteria and were included in the final review. No additional studies were identified through other sources, and no studies remained pending classification. The study selection process is summarized in Figure 1 (PRISMA flow diagram).

**Figure 1:**
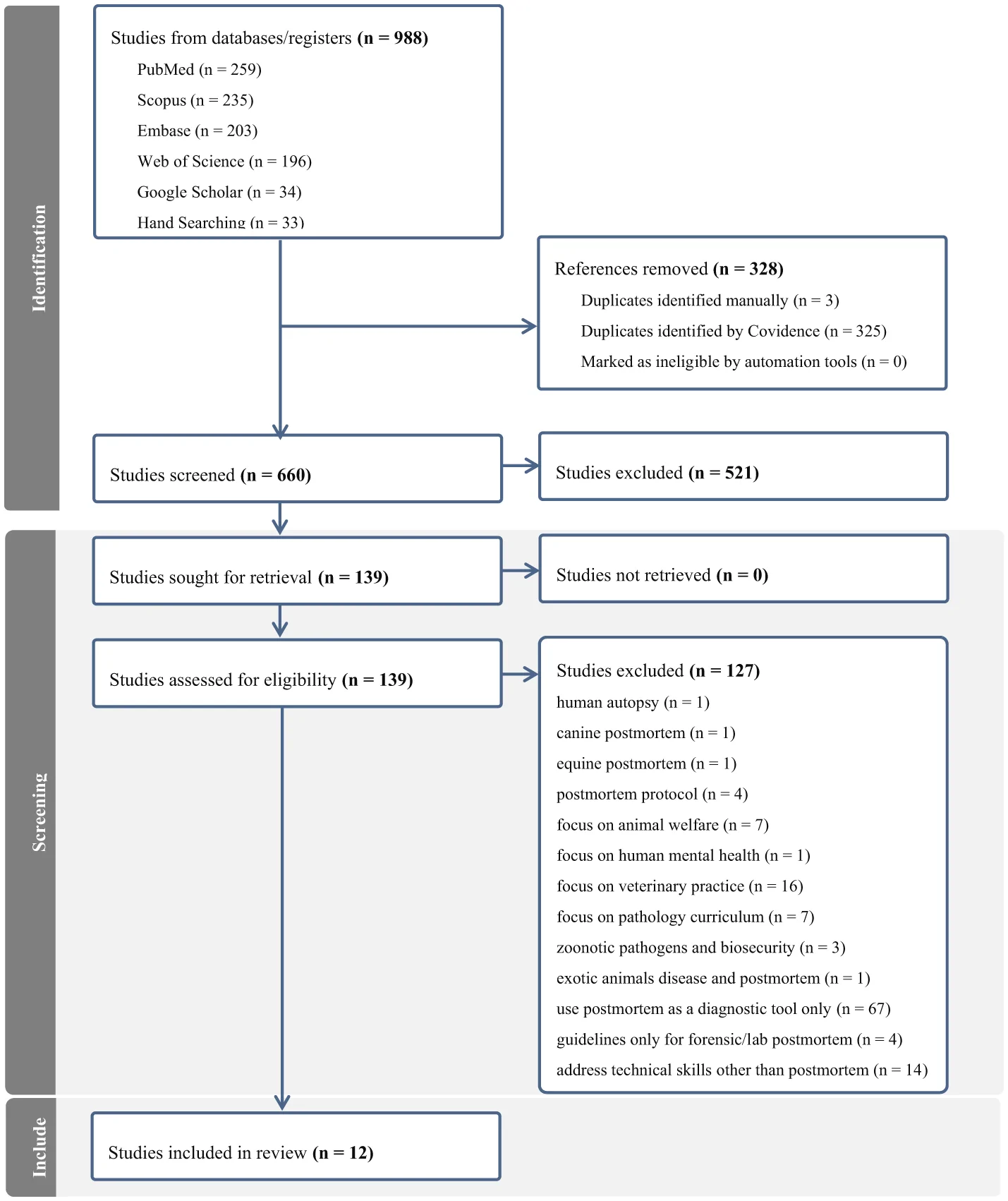
PRISMA flow diagram of the study identification, screening, eligibility, and inclusion process.

Based on the inclusion criteria, twelve references published between 2010 and 2024 were included in this scoping review. The studies were conducted across eight countries: five in the United States and one each in Nigeria, Sweden, the United Kingdom, Ireland, Denmark, Canada, and Brazil. Most studies used quantitative analytical approaches, while three combined quantitative and qualitative methods. Regarding study design, seven studies were cross-sectional surveys, three were observational studies, one was an experimental study, and one was a field-based pilot feasibility study with a qualitative component. The study populations involved a wide range of participants, including veterinarians, para-veterinarians (animal-health professionals with national diploma and/or higher national diploma in animal health), cattle producers, dairy farmers, and veterinary pathologists, as well as livestock populations. Animal populations primarily consisted of dairy and beef cattle with one study included horses, sheep, pigs, and goats in addition to cattle submitted for PME. Convenience sampling and systematic random sampling were the most frequently used sampling methods. Data were primarily collected through cross-sectional surveys administrated through online platforms such as Qualtrics, SurveyMonkey, or REDCap, an electronic data-capture platform used by the U.S. Centers for Disease Control and Prevention (CDC) or face-to-face. Six studies also incorporated training interventions, tissue sampling, photographic documentation, and laboratory diagnostic confirmation. Additional data sources included herd records, farmer interviews, and direct field observations.

## 4 Discussion

The literature evaluating field PME knowledge, skills, training, and practices among veterinary professionals and other relevant stakeholders involved in cattle production systems, including producers, farm workers, and managers is limited. During the screening process, most of the identified studies examined PMEs primarily as a diagnostic tool rather than investigating the knowledge, skills, or educational approaches required to perform field PMEs effectively. This finding highlights an important gap in the literature, indicating that considerably less attention has been given to the development and evaluation of educational strategies that support competency in field PMEs. Consequently, this lack of evidence may hinder the development of veterinary curricula, continuing professional development programs, and competency assessment frameworks related to field PMEs.

### 4.1 Knowledge and practical competencies in field PME

The included studies identified variation in knowledge and practical competencies relevant to field PME. The findings suggest that veterinarians possess theoretical knowledge required to perform PMEs, however, deficiencies remain in several practical competencies, including lesion interpretation, diagnostic sample collection, and the application of standardized biosecurity practices. These findings are supported by earlier evidence including 15, who reported that 92% of veterinarians correctly defined zoonoses, although 81% reported inadequate use of minimum PPE during PMEs. Likewise, 15 found agreement between remote and field diagnoses in seven of nine comparable cases; however, incomplete photographic examination contributed to a missed lesion. Davies et al. (12) reported that real-time pathologist assistance increased the proportion of field PMEs reaching a final diagnosis from 66 to 96% and supported appropriate tissue sampling, lesion recognition, and procedural consistency. Collectively, these studies suggest potential gaps in translating knowledge into consistent field PME practices.

Furthermore, these findings are also consistent with international veterinary competency frameworks, including those proposed by the World Organization of Animal Health (WOAH), which emphasizes the integration of multiple professional and diagnostic competencies. These include recognizing and interpreting gross lesions, applying diagnostic reasoning, collecting appropriate diagnostic samples, adhering to biosecurity protocols, producing accurate written and photographic documentation, and communicating effectively with producers to obtain relevant clinical histories and report findings (15). Importantly, theoretical knowledge of PMEs does not translate into competence in performing examinations under field conditions. Knowledge represents only one component of professional competence; effective field PMEs require the integration of technical skills, diagnostic reasoning, communication, and professionalism under authentic field conditions. This highlights the need for educational strategies that emphasize hands-on training, standardized PME protocols, continuing professional development, competency-based veterinary curricula, and the integration of emerging technologies to support documentation and remote consultation (12, 16, 17). Strengthening these components has the potential to improve PME quality, enhance diagnostic accuracy, reinforce disease surveillance, and promote adherence to biosecurity practices (18, 19). Accordingly, veterinary education should consider moving beyond theoretical knowledge and instead focus on demonstrating competence through simulated and world field-based conditions.

### 4.2 Perceptions of value and barriers to perform field PME

Overall, the included studies demonstrate positive perceptions and attitudes toward field PMEs among veterinarians. Field PMEs were generally perceived as valuable for disease diagnosis, herd health management, and disease surveillance. These perceived benefits were consistently reported in studies evaluating PME practices and educational interventions, in which veterinarians highlighted the importance of PMEs for improving diagnostic accuracy and obtaining information to support disease control and herd health decision-making.

Despite this strong recognition of PMEs' diagnostic value, their routine implementation remains inconsistent. The discrepancy suggests that positive attitudes alone are insufficient to ensure the systematic adoption of PMEs in cattle production systems. Rather, it highlights a gap between knowledge of recommended practices and their consistent application under field conditions. Several studies identified barriers to routine implementation, including producer reluctance, limited confidence in interpreting lesions, insufficient training in PME procedures and diagnostic sample collection, long distances to diagnostic laboratories, time constraints, and concerns related to biosecurity and zoonotic disease exposure. These findings suggest that recognition of the diagnostic value of PME does not necessarily translate into its consistent implementation under field conditions. Collectively, overcoming reported barriers may require educational approaches that go beyond teaching based solely on the transmission of knowledge, but rather incorporate practice through authentic field-based experiences, engagement in diagnostic decision-making, ensure exposure to an adequate caseload, receiving appropriate supervision, and structured feedback (20). Additional training in professional skills including communication, leadership and practice management can potentially minimize challenges associated with time, costs, and organizational support. Postmortem imaging may offer a complementary approach to traditional PME. Studies of veterinary virtopsy have explored modalities such as radiography, computed tomography, and magnetic resonance imaging to document internal abnormalities while preserving the external integrity of the carcass and potential forensic evidence (21). Similarly, pre-necropsy imaging has been shown to identify internal abnormalities and guide subsequent pathological examination and sample collection (22). Although these imaging approaches may not identify all lesions, they may provide valuable complementary diagnostic information. However, their feasibility and acceptability in commercial cattle field settings requires further evaluation. PME findings may also have legal or forensic relevance, particularly when PMEs are systematically documented and appropriate chain-of-custody procedures are followed.

### 4.3 Diagnostic practices and decision-making

The findings of this review suggest that the diagnostic value of field PMEs depends not only on the identification of lesions but also on the systematic integration of clinical history, standardized examination procedures, diagnostic sample collection, photographic and written documentation, laboratory analyses, and diagnostic decision-making. The development and implementation of standardized field PME protocols, supported by digital and emerging technologies, could assist veterinarians in determining when PME gross findings are sufficient to establish a presumptive diagnosis, and when additional testing is required to confirm the diagnosis (23). Standardized PME protocols can reduce variability among practitioners, promote thorough examination, and facilitate comparison of findings across cases and production systems.

Farmers and other stakeholders may also support the diagnostic process by providing information about the cause of death, clinical history, previous treatment, or other type of observations. Appropriately trained personnel can use standardized procedures to document relevant findings through photographs or real-time video for remote diagnosis by veterinarians. Likewise, digital photography and telepathology can enhance diagnostic accuracy by enabling remote consultations with veterinary pathologists when findings are uncertain. For example, 11 demonstrated that structured postmortem records integrating PME findings with clinical history, treatments, reproductive status, and management information can provide a more comprehensive understanding of the causes contributing to dairy cow mortality. Similarly, Lemos et al. (19) reported that real-time communication between field veterinarians and veterinary diagnostic laboratories through messaging applications facilitated the exchange of photographs, videos, and clinical epidemiological information, supporting diagnostic evaluation. However, digital tools should complement, rather than replace, systematic examination and sound diagnostic reasoning. By adopting standardized protocols, farm workers and veterinarians can ensure that all relevant organs are examined, findings are consistently documented, and appropriate diagnostic samples are collected before carcass disposal.

Accurate documentation may also have legal or forensic relevance. A retrospective study of 1,221 uncoded medical records demonstrated that structured diagnostic codes and standardized labeling can facilitate the identification and organization of cases with potential forensic relevance (24). In cattle production, accurate specimen collection, appropriate chain-of-custody procedures, comprehensive laboratory documentation, and professional interpretation may be particularly important in cases involving reproductive disease, abortion, zoonotic infection, suspected negligence, or disagreements over diagnostic findings, where legal or economic implications may arise (25).

### 4.4 Educational and training needs

The design, delivery, and educational framework of PME training programs should be targeted to the learning needs and responsibilities of each stakeholder group in the cattle production industry, while also considering differences in professional experience. Evidence from the studies included supports the need for stakeholders-specific training. For example, early-career bovine veterinarians reported a preference for educational programs that combine online learning with hands-on practical workshops (17). In contrast, practicing dairy veterinarians identified continuing education needs in areas such as neurologic diseases, interpretation of gross lesions, and recognition of disease-patterns (2). Para-veterinarians reported lower levels of knowledge regarding zoonotic diseases and lower risk perception during PMEs and tissue collection, highlighting the need for targeted training in biosecurity and zoonotic disease prevention (17).

Future educational programs should integrate theoretical knowledge with the practical competencies required to effectively perform field PMEs and support training in lesion recognition, diagnostic reasoning, diagnostic sample collection, standardized documentation, biosecurity, and effective communication with producers and diagnostic laboratories. McConnel et al. (11) and Lemos et al. (19) highlighted the need to train veterinarians, diagnostic laboratory personnel, and farm personnel in the systematic conduct and documentation of PMEs. For veterinarians, training should emphasize standardized necropsy procedures, accurate lesion recognition and documentation, and appropriate sample collection. In addition, emerging educational technologies can offer opportunities to enhance field PME training. Specifically, telepathology consultations, mobile applications, AI-assisted diagnostic decision support systems, and online learning platforms can improve access to training, facilitate standardized documentation, and promote greater diagnostic consistency, particularly in geographically remote areas (26). Collectively, these findings support a shift toward competency-based PME education that integrates foundational knowledge with deliberate practice in authentic field settings.

Overall, the available evidence indicates that field PMEs are valuable for diagnosis, herd-health management, and disease surveillance. However, their consistent implementation is limited by gaps in practical training, lesion recognition and interpretation, diagnostic sampling, documentation, biosecurity, and access to laboratory support. The current literature provides limited evidence regarding the frequency with which field PMEs are conducted, the organs routinely examined, the types and quality of samples collected, the influence of veterinarian capacity and availability of resources regarding PMEs. Furthermore, it remains unclear which educational approaches lead to sustained improvements in veterinarian competence. Evidence regarding the feasibility and effectiveness of postmortem imaging, digital decision support tools, and AI-assisted interpretation in commercial cattle settings also remains limited.

### 4.5 One health implications

These findings carry important implications within the One Health framework. Beyond guiding herd-level interventions and individual postmortem diagnoses, field PMEs can contribute to the detection and surveillance of emerging infectious and zoonotic diseases in cattle. When systematic lesion recognition and rigorous sample collection are combined with standardized documentation, laboratory confirmation and reporting, field PMEs may strengthen early-warning surveillance of health threats at the human-animal interface.

However, this review also identifies systemic vulnerabilities related to occupational and public health. Inconsistent use of PPE, together with gaps in awareness of zoonotic risks among veterinarians and farm workers, may increase occupational exposure during field PMEs. Addressing these risks requires rethinking field PME training that integrates biosecurity, biosafety, zoonotic disease recognition, and appropriate public health reporting alongside diagnostic pathology. Strengthening these competencies may enhance both the diagnostic value of field PMEs and their contribution to One Health surveillance and preparedness.

### 4.6 Limitations

We acknowledge that this study is limited only to references published in English between 2000 and 2025. Additionally, seven of the twelve included studies reported cross-sectional self-reported survey data which inherently include potential reporting bias, recall bias, and social desirability bias. Furthermore, only three out of twelve studies incorporated training, educational or structured intervention. The scarcity of evidence limits our ability to draw definitive, evidence-based conclusions on which specific pedagogical approaches are most effective for different stakeholder groups, veterinarians, para-veterinarians, and producers. Another limitation of the study relates to the heterogeneity of statistical methodology followed in the selected studies and resulting challenges in the synthesis of data. Lastly, 11 out of the 12 included studies were conducted in the Global North, defined here as high-income countries primarily located in North America and Europe, with only two studies conducted in Latin America and Africa (Brazil and Nigeria, respectively). This can represent a major geographical bias, considering the existing realities of veterinary infrastructure, access to diagnostic laboratories, and economic barriers differ vastly in low-resource settings. Consequently, this geographical concentration may limit the applicability of the findings to lower-resource settings and countries.

## 5 Conclusion

This review identified limited evidence regarding the competencies and training required for field PMEs in cattle production systems, with the existing literature placing greater emphasis on the diagnostic value of PMEs than on how these competencies are taught or assessed. Although veterinary professionals recognize the value of field PMEs, gaps and inconsistencies remain in lesion interpretation, sample collection, biosecurity practices, documentation, and diagnostic decision-making. These findings support the need for standardized protocols, and competency-based curricula that integrate theoretical knowledge with practical skill development. Educational approaches that integrate online learning with practical hands-on training and are tailored to the needs of specific stakeholder groups may further enhance competency development. Emerging digital technologies, including photographic documentation and remote consultations with veterinary pathologists, can complement these educational strategies by improving diagnostic support without replacing the value of systematic PMEs. Future studies are needed to develop targeted competency-based educational interventions and determine their impact on field PME outcomes. Ultimately, strengthening competency-based field PME training has the potential to improve diagnostic quality, support disease surveillance, and enhance herd health management across cattle production systems.

## Data Availability

The original contributions presented in the study are included in the article/Supplementary material, further inquiries can be directed to the corresponding author.

## Supplementary material

Supplementary Table S1 Summary of the characteristics and extracted data from the studies included in the scoping review.
